# Analysis of Insulin Doses of Chinese Type 2 Diabetic Patients with Intensive Insulin Treatment

**DOI:** 10.1371/journal.pone.0038962

**Published:** 2012-06-18

**Authors:** Xiaoling Cai, Xueyao Han, Yingying Luo, Linong Ji

**Affiliations:** Endocrinology and Metabolism Department, Peking University People’s Hospital, Beijing, China; Pennington Biomedical Research Center, United States of America

## Abstract

**Background:**

To investigate the daily insulin doses and the ratio of basal insulin to total daily insulin in Chinese type 2 diabetic patients who received basal bolus insulin therapy.

**Methodology/Principal Findings:**

Totally 2480 patients prescribed with pre-meal bolus insulin and bedtime basal insulin were included. The mean daily insulin doses was 38.22±14.92 IU/day, the mean daily insulin doses per weight was 0.58±0.22 IU/kg, the mean bolus insulin dose was 0.44±0.17 IU/kg and the mean basal insulin dose was 0.13±0.08 IU/kg. The mean basal/total daily insulin ratio (BD/TDD) was 0.23±0.08. In most patients (47.94%), the BD/TDD was between 0.20 and 0.30. Diabetic duration, BMI, HbA1c, fasting and postprandial blood glucose level were positively associated with daily insulin dose, while age was negatively associated with daily insulin dose. Diabetic duration, BMI, HbA1c, fasting blood glucose level, and using metformin were positively associated with BD/TDD ratio, while age, postprandial C peptide, postprandial blood glucose level and CRE level were negatively associated with BD/TDD ratio.

**Conclusions/Significance:**

The daily insulin doses of intensive treatment in Chinese type 2 diabetic patients was 38.22 IU/day, the mean daily insulin doses per weight was 0.58 IU/kg, mean BD/TDD ratio was 0.23.

## Introduction

Type 2 diabetes is a progressive disease. With the advance of the disease, many patients will need insulin to maintain good glycemic control in order to minimize the risk of diabetic complications. For patients inadequately controlled by oral antihyperglycemic agents, basal insulin is recommended to be added to the oral agent [1], [2]. When glycemic control can no longer be achieved or maintained with this therapy, then prandial insulin is gradually added, leading to a final basal/bolus treatment (BBT) for insulin intensification [3]–[5].

In clinical practice, insulin treatment needs to be highly individualized in terms of doses and regiment in order to reach a balance between good glycemic control and the risk of hypoglycemia. In theory, the BBT is the ideal insulin treatment regiment for individualized insulin treatment because of its timing of insulin injection and adjustment is more physiological. However, what is the proper insulin dosage of BBT and what are the associated factors in Chinese type 2 diabetic patients are still not clear. Moreover, what is the proper basal insulin to total daily insulin dose ratio and the associated factors in Chinese type 2 diabetic patients are yet to be determined.

To assess the proper insulin dosage and proper basal insulin to total daily insulin dose ratio in Chinese type 2 diabetic patients, we made this retrospective analysis in hospitalized patients receiving intensive insulin treatment by BBT.

## Methods

### 1. Patients

By searching inpatient database at Peking University People’s Hospital, we have identified 2480 type 2 diabetic patients who were hospitalized for BBT treatment due to the poor glycemic control at the ward of Department of Endocrinology and Metabolism of Peking University People’s Hospital from Jan. 2005 to Dec. 2010. Reasons for BBT were as followings: 1) Advancement of insulin treatment from basal insulin plus oral hypoglycemic agent treatment or from treatment with only oral hypoglycemic agents before. 2) Advancement of insulin treatment from premixed insulin when blood glucose could not be adequately controlled. 3) Optimizing the BBT. Patients with diabetic ketoacidosis or ketonuria and tested positive for anti-glutamic acid decarboxylase and islet cell antibody were excluded. Patients prescribed other oral hypoglycemia agent except metformin were excluded.

### 2. Ethics Statement

This is a retrospective study, and the data were analysed anonymously, therefore, there is no need for informed consent. The ethics committee of Peking University People’s Hospital has approved this retrospective study. All the patients were patients admitted in the department of Endocrine & Metabolism of our hospital, and during the day of their admitted, they signed the consent form for allowing their information to be stored in the hospital database and used for research, and this consent form was also approved by the ethics committee of Peking University People’s Hospital.

### 3. Insulin Initiation and Titration

According to the BBT treatment protocol of our department, each patient admitted received insulin regimen as bolus insulin (human insulin, Humilin R, Illy Lilly) and basal insulin (neutral protamine Hagedorn [NPH] insulin, Humilin N, Illy Lilly). The BBT treatment protocols were shown as follows:

- For patients with advancement of insulin treatment from basal insulin plus oral hypoglycemic agent treatment or from treatment with only oral hypoglycemic agents before: 1) Start basal-bolus regimen with NPH and human regular insulin. 2) Discontinue oral antidiabetic drugs except metformin on admission. 3) Start total daily insulin dose as 0.4 units/kg of body weight/day. 4) Give one-third of total daily dose as basal insulin (NPH) and two-third as bolus insulin(human regular insulin). 5) The bolus insulin was injected 30 minutes before each meal and the basal insulin was injected around 10 pm. 6) Each patient was measured for fasting and two hour post-prandial glucose (2 hPBG) after each meal every day. 7) The bolus insulin doses were adjusted according to 2 hPPG following insulin injection by 2∼4 units per day. The basal insulin doses were adjusted according to fasting glucose level (FBG) of previous day by 2 units per day. 8) The goal of fasting glucose control level is 4.0∼6.0 mmol/L and of 2 hPBG control is 5.0∼8.0 mmol/L. 9) If patient develops hypoglycemia (<4.0 mmol/l) or has symptoms of hypoglycemia, decrease NPH or regular insulin dose by 2 units according to fasting glucose level or 2 hPBG.
- For patients with advancement of insulin treatment from premixed insulin when blood glucose could not be adequately controlled: 1) Discontinue premixed insulin and start basal-bolus regimen with NPH and human regular insulin. 2) Discontinue oral antidiabetic drugs except metformin on admission. 3) Start total daily insulin dose which was calculated according to total insulin dose of premixed insulin before admitted. Step 4 to 9 were as the above shown.
- For patients with optimizing the BBT: 1) Continue the total insulin dose of basal-bolus regimen before admitted. 2) The bolus insulin doses were adjusted according to 2 hPBG following insulin injection by 2∼4 units per day. The basal insulin doses were adjusted according to fasting glucose level of previous day by 2 units per day. 3) The goal of fasting glucose control level is 4.0∼6.0 mmol/L and of 2 hPBG control is 5.0∼8.0 mmol/L. 4) If patient develops hypoglycemia (<4.0 mmol/l) or has symptoms of hypoglycemia, decrease NPH or regular insulin dose by 2 units according to fasting glucose level or 2 hPBG. 5) If the patients were overweight and could tolerate metformin, doctors would prescribe metformin for better glycemic control.

Among these patients, 1119 patients received insulin therapy alone while 1361 patients received insulin combination with metformin at a standard dose 1500 mg/day. During hospitalization, patients were prescribed standard diabetic meal comprising 60 percent of carbohydrate, 25 percent of fat and 15 percent of protein. The mean duration of hospital stay was 14±2.4 days.

### 4. Biochemical and Clinical Measurements

In addition to the details of insulin treatment, data retrieved from inpatient database included anthropometric measurements at admission, body weight of the first day and the last day of hospitalization were measured, 4 points capillary glucose profiles (fasting, two hours post-breakfast, two hours post-lunch and two hours post-supper) were measured by glucose meters (ACCU-CHEK®, Advantage, Roche Diagnostic) during hospitalization, biochemical measurements included fasting and 2 hour postprandial C-peptide (ACS180,BYAER, Fribourg, Switzerland), HbA1c (Primus ultra2, Primus Diagnostics, MO, USA ).

### 5. Statistical analysis

Since the patients were admitted into the ward and discharged from hospital at different time of first day and last day of hospitalization, the data of insulin doses, fasting and postprandial glucose were sporadic at first day and last day of hospitalization. Therefore, the analyses of those data were based on the insulin doses, 4 points glucose profile of second day and the second to last day of hospitalization. The postprandial glucose was expressed as the mean of three postprandial glucose levels of the day. Analyses of insulin dose were based on insulin doses of basal and prandial insulin on the last day of hospitalization.

All values are represented as means ± s.d. Continuous variables were compared using a two-sample t test while frequency of dichotomous variables was performed by χ^2^ analysis. A two-sided p≤0.05 was considered significant. Multiple linear regression analysis was used to assess the associated factors of insulin doses. All the data was analyzed by using SPSS19.

## Results

### 1. Characteristics and Glucose Control during Hospitalization of these Patients

Clinical characteristics of patients were shown in Table 1. The average age was 58.87 years old; the average diabetic duration was 11.3 years. The average HbA1c was 9.41%. When these patients were admitted, the average fasting glucose level was 8.51 mmol/L and the average 2 hr PBG was 13.35 mmol/L. When these patients were discharged from hospital, the average fasting glucose level and 2 hPPG level was 7.28 mmol/L and 8.9 7 mmol/L respectively. Details were shown in Table 1. Clinical characteristics of patients were also shown in Table 1.

**Table 1:** Demographics and characteristics of type 2 diabetic patients receiving BBT.

Variable	All patients	Patients receiving insulintherapy alone	Patients receiving bothinsulin and metformin
**N**	**2480**	**1119**	**1361**
**Male/female**	**1337/1143**	**630/489**	707/654*
age (years)	58.87±13.15	58.59±14.09	59.11±12.32
Duration of DM (years)	11.30±7.96	10.61±8.37	11.87±7.56
BMI (kg/m^2^)	24.53±3.59	23.31±3.49	25.53±3.35
Waist hippo ratio (WHR)	0.91±0.09	0.90±0.11	0.92±0.07
SBP (mmHg)	133.31±19.38	132.71±20.93	133.80±18.01
DBP (mmHg)	78.30±10.58	77.82±10.84	78.68±10.36
HbA1c (%)	9.41±2.15	9.46±2.43	9.37±1.91
Fasting C-peptide (ng/dL)	1.56±0.91	1.44±0.96	1.65±0.85
postprandial C-peptide (ng/dL)	3.16±2.10	2.99±2.24	3.28±1.98
FBG (mmol/L) when admission	8.51±3.00	8.21±3.02	8.75±2.96
2 hPBG (mmol/L) when admission	13.35±5.07	13.38±5.56	13.33±4.66
FBG (mmol/L) Discharged from hospital	7.28±1.84	7.36±2.05	7.22±1.65
2 hPBG (mmol/L) Discharged from hospital	8.97±2.48	9.10±2.67	8.86±2.31
CRE (umol/L)	78.68±40.02	85.88±54.50	73.18±22.11
bolus Insulin dose per day (IU/day)	29.29±11.31	27.67±11.25	30.61±11.18
Basal Insulin dose per day (IU/day)	8.94±5.33	7.77±4.61	9.91±5.68
Total Insulin dose per day (IU/day)	38.22±14.92	35.44±14.27	40.51±15.06
Bolus Insulin doses per kilogram (IU/kg)	0.44±0.17	0.44±0.18	0.44±0.16
Basal Insulin doses per kilogram (IU/kg)	0.13±0.08	0.12±0.07	0.14±0.08
Total Insulin doses per kilogram (IU/kg)	0.58±0.22	0.57±0.23	0.59±0.22
Pre-breakfast Insulin doses per kilogram (IU/kg)	0.17±0.07	0.17±0.07	0.17±0.07
Pre-lunch Insulin doses per kilogram (IU/kg)	0.12±0.06	0.12±0.06	0.12±0.05
Pre-supper Insulin doses per kilogram (IU/kg)	0.15±0.06	0.15±0.07	0.15±0.06
BD/TDD ratio	0.23±0.08	0.22±0.09	0.24±0.08

### 2. Insulin Doses and Associated Factors

The mean daily insulin dose was 38.22±14.92 IU. The mean insulin dose per weight was 0.58±0.22 IU/kg, among which, the mean bolus insulin dose was 0.44±0.17 IU/kg and the mean basal insulin dose was 0.13±0.08 IU/kg. Of the bolus insulin dose, the pre-breakfast one was 0.17±0.07 IU/kg, the pre-lunch one was 0.12±0.06 IU/kg, and the pre-dinner one was 0.15±0.06 IU/kg, representing 38.6%, 27.3% and 34.1% of bolus insulin dose respectively. Subgroup analysis of patients with insulin therapy alone and patients with insulin combination with metformin therapy showed that, compared with patients treated with insulin and metformin, patients treated with insulin alone showed lower daily insulin doses (35.44±14.27 vs. 40.51±15.06 IU/day, p = 0.00), lower bolus insulin dose (27.67±11.25 vs. 30.61±11.18 IU/day, p = 0.00), lower basal insulin dose (7.77±4.61 vs. 9.91±5.68 IU/day, p = 0.00). And when it turns to insulin doses per kilogram of body weight, patients with insulin therapy alone showed lower insulin doses per weight (0.57±0.23 vs. 0.59±0.22 IU/kg, p = 0.00), and lower basal insulin doses per weight (0.12±0.07 vs. 0.14±0.08 IU/kg, p = 0.00). (shown in Table 1.).

Then, multiple linear regression analysis was used to find the associated factors with insulin dose. Insulin doses per day was used as dependent variable and diabetic duration, age, BMI, w/r, SBP, DBP, HbA1c, fasting and postprandial C peptide, FBG and 2 hrPBG level when admission and discharged from hospital, creatinine level and whether or not using metformin as independent variables. Results showed that diabetic duration, BMI, HbA1c, FBG and 2 hrPBG level when admission, FBG level when discharged from hospital were independent associated factors and positively with daily insulin dose, while age was negatively associated with daily insulin dose, shown in Table 2. Subgroup analysis of patients with insulin therapy alone and patients with insulin combination with metformin therapy showed similar associated factors, also shown in Table 2.

**Table 2:** Factors associated with BD/TDDratio, insulin doses per day and insulin doses per kilogram of body weight (revised by gender) in all patients, insulin alone treated patients and insulin combination with metformin treated patients.*.

Variable	all patients			insulin alone treated patients			insulin combination with metformin treated patients		
	BD/TDD ratio	insulin dosesper day	insulin dosesper kilogram	BD/TDD ratio	insulin dosesper day	insulin dosesper kilogram	BD/TDD ratio	insulin dosesper day	insulin dosesper kilogram
age	−0.164	−0.179	−0.145	/	−0.225	−0.183	−0.183	−0.173	−0.127
DM duration	0.102	0.276	0.259	/	0.266	0.244	0.143	0.271	0.244
BMI	0.152	0.282	−0.117	0.177	0.306	/	0.151	0.218	−0.137
HbA1c	0.129	0.21	0.23	/	0.207	0.243	0.132	0.213	0.208
Fasting C peptide	/	/	/	/	/	/	/	/	/
Postprandial C peptide	−0.111	/	/	−0.204	/	/	−0.082	/	/
FBG (Admission)	0.12	0.067	0.079	/	/	/	0.153	/	/
2 hrPBG (Admission)	/	0.076	0.067	/	/	/	/	0.112	0.107
FBG (Discharged from hospital)	0.127	0.151	0.126	/	/	/	0.18	0.204	0.157
2 hrPBG (Discharged from hospital)	−0.117	/	/	−0.124	0.096	0.119	−0.124	/	/
CRE(umol/L)	−0.131	/	/	/	/	/	−0.113	/	/
Metformin	0.092	/	/	/	/	/	/	/	/

* The value was described as β values in multiple linear regression analysis (p<0.01).

And when it turns to insulin dose per kilogram of body weight, results showed that diabetic duration, HbA1c, FBG and 2 hrPBG level when admission, FBG level when discharged from hospital were independent associated factors and positively with daily insulin dose per weight, while age, BMI were negatively associated with insulin dose per weight, shown in Table 2. Subgroup analysis showed that the associated factors were similar.

For further analysis, the patients were divided according to the tertiles of those above associated factors, the average level of each associated factor in each group of patients was shown in Table 3. From this analysis, we can learn more details. Such as, according to the tertiles of diabetic duration, patients were divided into three groups as diabetic duration≤7 years, between 8 and 14 years, ≥15years. Daily insulin doses in these three groups of patients were 35.60±13.63, 38.87±14.80 and 40.30±15.88 IU/day respectively, and insulin dose per weight in these three groups of patients were 0.53±0.21, 0.58±0.22 and 0.62±0.23 IU/kg respectively.

**Table 3:** The BD/TDD ratio and insulin dose per kilogram of body weight were then analyzed by stratifying the study subject with age, DM duration, BMI, HbA1c, C peptide level, fasting and 2h glucose level on admission and discharged from hospital, and usage of metformin.

Variables		BD/TDD ratio	insulin doses perkilogram of body weight	Total insulin doseper day
Age*	≤53 years	0.24±0.09	0.57±0.21	38.96±14.25
	54–66 years	0.23±0.08	0.59±0.23	39.75±16.14
	≥67 years	0.21±0.08	0.57±0.22	35.90±14.04
DM duration	≤7years	0.22±0.08	0.53±0.21	35.60±13.63
	8–14years	0.23±0.09	0.58±0.22	38.87±14.80
	≥15years	0.23±0.09	0.62±0.23	40.30±15.88
BMI*	<22.2 kg/m^2^	0.21±0.09	0.63±0.23	34.41±12.33
	22.2–25.8 kg/m^2^	0.22±0.08	0.56±0.21	36.89±13.66
	≥25.9 kg/m^2^	0.24±0.09	0.56±0.22	43.40±16.99
HbA1c	≤8.2%	0.22±0.09	0.52±0.20	34.65±13.99
	8.3–10.1%	0.23±0.08	0.60±0.24	40.66±16.14
	≥10.2%	0.24±0.08	0.62±0.21	39.98±13.61
fasting C-peptide	<1.18 ng/dl	0.23±0.08	0.59±0.21	37.42±12.54
	1.18–2.04 ng/dl	0.23±0.08	0.59±0.21	40.32±15.23
	≥2.05 ng/dl	0.22±0.08	0.58±0.23	40.98±16.30
Postprandial C-peptide	<1.89 ng/dl	0.24±0.08	0.62±0.21	38.42±12.91
	1.89–3.48	0.24±0.08	0.61±0.22	41.84±15.13
	≥3.49	0.22±0.07	0.55±0.22	38.87±15.84
FBG (mmol/L) when admission *	<6.95	0.21±0.08	0.53±0.20	35.26±14.07
	6.95–9.39	0.23±0.08	0.59±0.22	39.39±14.41
	≥9.4	0.25±0.08	0.64±0.23	42.66±15.15
2 hrPBG (mmol/L) when admission *	<11.1	0.22±0.09	0.54±0.20	35.98±13.69
	11.1–15	0.23±0.09	0.59±0.23	39.97±16.10
	≥15.1	0.24±0.08	0.63±0.22	41.34±14.43
FBG (mmol/L) Discharged from hospital	<6.4	0.22±0.08	0.55±0.21	36.12±13.18
	6.4–7.5	0.22±0.08	0.51±0.21	38.51±14.32
	≥7.6	0.24±0.09	0.62±0.24	40.55±16.53
2 hrPBG (mmol/L) Discharged from hospital *	<7.8	0.24±0.08	0.58±0.22	37.80±14.03
	7.8–9.2	0.22±0.08	0.57±0.22	38.11±14.02
	≥9.3	0.21±0.08	0.59±0.23	39.43±15.78
Usage of metformin*	Insulin alone	0.22±0.09	0.57±0.23	35.44±14.27
	Insulin+metformin	0.24±0.08	0.59±0.22	40.51±15.06
Creatinine (umol/L)	<66	0.24±0.08	0.61±0.22	40.13±15.32
	66–83	0.23±0.08	0.58±0.22	38.58±14.63
	≥84	0.22±0.07	0.54±0.22	37.12±14.86

* P values among the three groups were less than 0.05.

### 3. Ratio between Basal Insulin Doses and Total Daily Insulin Doses and Associated Factors

In all patients, the ratio between basal insulin doses and total daily insulin doses (BD/TDD ratio) was 0.23±0.08 (shown in Table 1). Subgroup analysis showed that compared with patients using insulin combination with metformin therapy, patients with insulin therapy alone showed significantly lower BD/TDD ratio (0.22±0.09 vs 0.24±0.08, P<0.05).

Then, multiple linear regression analysis was also used to find the associated factors with BD/TDD ratio. Results showed that diabetic duration, BMI, HbA1c, fasting blood glucose level when admission and when discharged from hospital, and using metformin were the independent associated factors and positively with BD/TDD ratio, while age, postprandial C peptide, postprandial blood glucose level when discharged from hospital, and creatinine level were negatively associated with BD/TDD ratio, shown in Table 2. Subgroup analysis showed similar results.

The BD/TDD was then analyzed by stratifying the study subjects with each associated factor such as age, diabetic duration, BMI, HbA1c, C peptide level, fasting glucose and 2 hr PPG level, and usage of metformin (Table 3).

### 4. Distribution of BD/TDD in Patients

**Figure 1:**
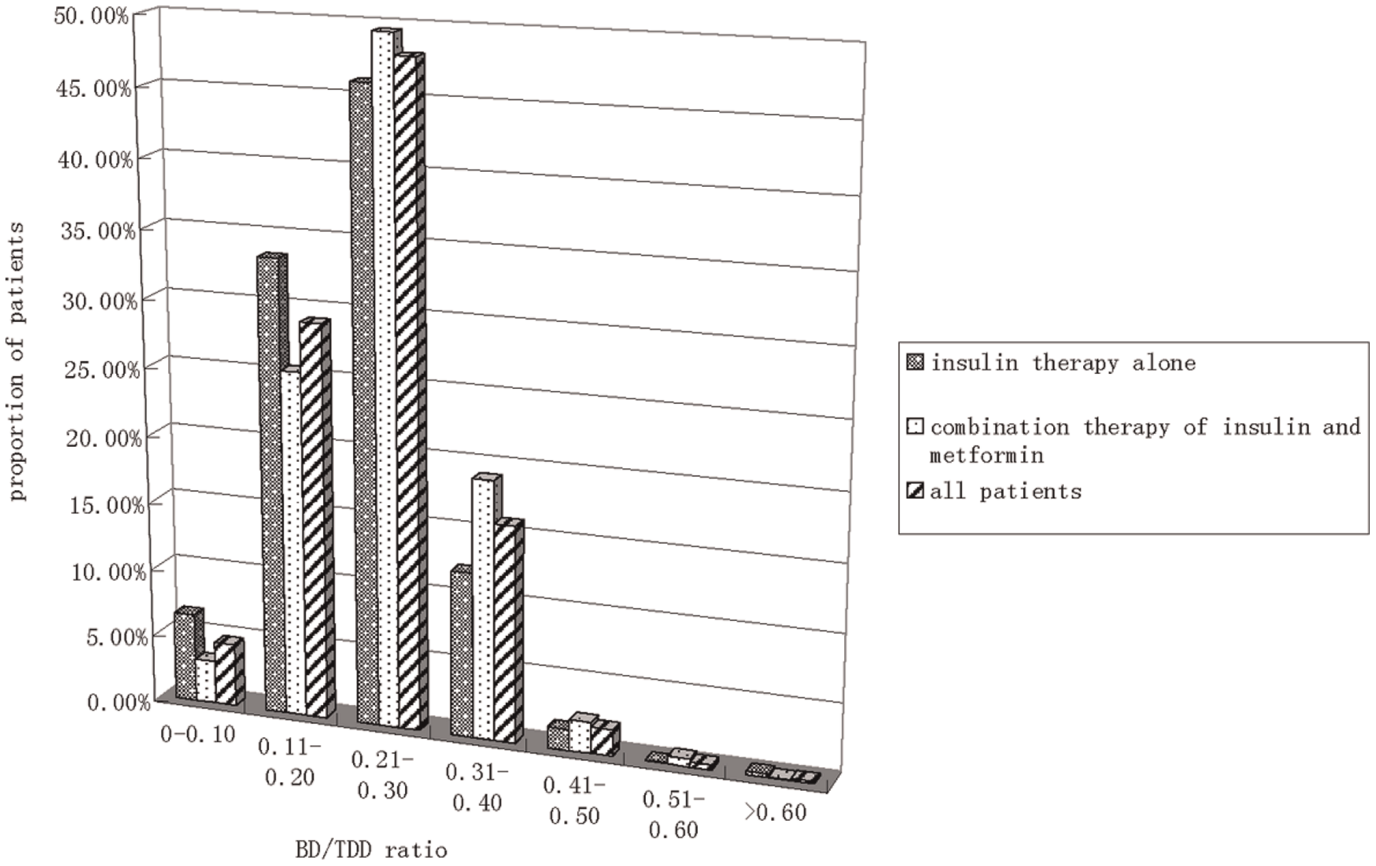
The distribution of BD/TDD of type 2 diabetic patients receiving intensive insulin therapy. Patients whose BD/TDD ratio between 0 and 0.10 occupied 4.64%, patients whose BD/TDD ratio between 0.11 and 0.20 occupied 29.03%, patients whose BD/TDD ratio between 0.21 and 0.30 occupied 47.94%, patients whose BD/TDD ratio between 0.31 and 0.40 occupied 15.93%, patients whose BD/TDD ratio between 0.41 and 0.50 occupied 1.94%, patients whose BD/TDD ratio between 0.51 and 0.60 occupied 0.36%, patients whose BD/TDD ratio more than 0.60 occupied 0.16%. Subgroup analysis showed that: of the patients receiving insulin therapy alone, Patients whose BD/TDD ratio between 0 and 0.10 occupied 6.52%, patients whose BD/TDD ratio between 0.11 and 0.20 occupied 33.42%, patients whose BD/TDD ratio between 0.21 and 0.30 occupied 46.02%, patients whose BD/TDD ratio between 0.31 and 0.40 occupied 12.15%, patients whose BD/TDD ratio between 0.41 and 0.50 occupied 1.52%, patients whose BD/TDD ratio between 0.51 and 0.60 occupied 0.09%, patients whose BD/TDD ratio more than 0.60 occupied 0.27%. Of the patients receiving insulin combination with metformin therapy, patients whose BD/TDD ratio between 0 and 0.10 occupied 3.16%, patients whose BD/TDD ratio between 0.11 and 0.20 occupied 25.40%, patients whose BD/TDD ratio between 0.21 and 0.30 occupied 49.49%, patients whose BD/TDD ratio between 0.31 and 0.40 occupied 19.02%, patients whose BD/TDD ratio between 0.41 and 0.50 occupied 2.28%, patients whose BD/TDD ratio between 0.51 and 0.60 occupied 0.56%, patients whose BD/TDD ratio more than 0.60 occupied 0.07%. The three columns represented patients receiving insulin alone, patients receiving insulin combination with metformin and all of the patients, respectively, from left to right.

Figure 1 showed the distribution of BD/TDD ratio in the studied population in both numerically and graphic manner. The number of patients whose BD/TDD ratio between 0.11 and 0.40 occupied 92.9%, while the number of patients whose BD/TDD ratio no more than 0.10 occupied 4.64%, and the number of patients whose BD/TDD ratio more than 0.40 occupied 2.46%. In the patients with insulin thearpy alone and patients with insulin combination with metformin, the pattern of distribution of the BD/TDD ratio was nearly the same. (Figure 1).

## Discussion

In this study, we used insulin dose information from type 2 diabetic patients hospitalized for BBT to analysis daily insulin dose and the ratio between basal insulin (NPH) and total insulin dose (NPH +short acting insulin), with the aim of estimating daily requirement for basal and preprandial insulin for BBT and evaluating the suitable basal/bolus ratio for intensive insulin treatment. In addition, factors associated with insulin doses and basal/bolus ratio were also analyzed. What unique of this study is that insulin doses collected for analysis in this study was the doses associated with good glycemic control (with average fasting glucose level 7.28±1.84 mmol/L and 2 hr postprandial glucose level 8.97±2.48 mmol/L when discharged from hospital, and without severe hypoglycemia) during hospitalization.

From this study, we concluded that average daily insulin dose of Chinese type 2 diabetic patients receiving BBT was 38.22 IU/day (0.58 IU/kg/day). Average basal insulin dose was 8.94 IU/day (0.13 IU/kg/day) and average bolus insulin dose was 29.29 IU/day (0.44 IU/kg/day). Of the patients receiving insulin therapy alone or receiving insulin combination with metformin, daily insulin dose was 35.44 IU/day (0.57 IU/kg/day) and 40.51 IU/day (0.59 IU/kg/day) respectively. The insulin dose of Chinese type 2 diabetic patients in our study may be different from that reported in the Caucasians. Rosenstock et al reported in a study [6] that eligible subjects had type 2 diabetes inadequately controlled by previous insulin therapy plus OHAs, an average duration of diabetes of >10 years, and baseline BMI of 36.7 kg/m2, and the daily insulin dose of BBT was 146 IU/day (1.4 IU/kg/day), among which the basal insulin doses was 54.9 IU/day (0.56 IU/kg/day). Hollander et al [7]reported in a 52-week study of BBT comparing detemir combination with aspart to glargine combination with aspart, in the detemir group, daily detemir and aspart doses were 0.82 U/kg and 0.36 U/kg, respectively and total daily dose was 1.18 U/kg, while in the glargine group, daily glargine and aspart doses were 0.59 and 0.32 U/kg, respectively, and total daily dose was 0.91 U/kg. Umpierrez et al [8] compared BBT (glargine/glulisine) plus OHAs to sliding-scale regular insulin (SSI) in the inpatient management of with type 2 diabetic patients, mean daily insulin dose was about 42 U. Another comparison they made [9] was of inpatient insulin regimens with detemir plus aspart versus NPH plus regular in medical patients with type 2 diabetes. Mean total daily insulin dose in the detemir/aspart group was 57±45 U, mean daily dose of detemir insulin given once daily was 30±28 U, total daily dose of aspart insulin given before meals was 27±20 U. Fonceca et al [10] made a comparison between glargine with regular insulin and NPH with regular insulin, which showed that in the glargine group, daily glargine and regular insulin doses were 36.4±26.5 U and 37.1±28.4 U, respectively, total daily dose was 73.5 U, in the NPH group, daily NPH and regular insulin doses were 30.2±22.8 U and 34.0±24.1U, respectively, and total daily dose was 64.2 U. Raskin et al [11] compared insulin regimens with detemir plus aspart versus glargine plus aspart, which showed that the mean basal insulin doses were 0.81±0.456 U/kg and 0.75±0.324 U/kg for the detemir group and the glargine group, respectively. Insulin doses (including prandial and basal insulin doses) of all the above studies were much higher than that concluded from our study. There may be some reasons for the differences. First, the racial differences might explain the heterogeneity of insulin secretion and insulin sensitivity between Chinese and Caucasians, which may resulted in different insulin dosage. As we know that, daily doses of BBT reported from Japanese studies were comparable to that in Chinese. Yokoyama et al [12] compared BBT (glargine with aspart/lispro) to BBT (NPH with aspart/lispro), mean total daily insulin dose in the glargine group was 42±18 U, the percentage of basal insulin was 48%, while in the NPH group, mean total daily insulin dose was 38±16 U, the percentage of basal insulin was 28%. Other studies in Japanese patients showed that total daily insulin plus OHAs ranged from 0.31 IU/kg to 0.62 IU/kg [13], [14], [15]. Second, this study is a retrospective study while those studies in the Caucasians are random control studies or cross-sectional studies. There might be different protocols for insulin initiation and titration, different combination drugs and different target of glycemic control. Therefore, there might be different insulin dose and different BD/TDD ratio. Third, the patients included were all admitted patients, there might be different lifestyle intervention, different severity of the disease, different diabetic duration, compared with the patients in the Caucasians studies. Therefore, there might be different insulin dose and different BD/TDD ratio. However, reasons are still not clear at all.

According to this study, we found that patients prescribed with higher daily insulin doses showed lower age, longer diabetic duration, higher BMI, higher HbA1c, higher blood glucose level. And when it turns to insulin dose per kilogram of body weight, patients prescribed with higher insulin doses per weight also showed the above characteristics but different in lower BMI. In patients with insulin therapy alone, lower age, longer diabetic duration, higher BMI, higher HbA1c, higher blood glucose level were also associated with higher daily insulin dose, and the same factors without BMI were associated with higher insulin doses per weight. In patients with insulin combination with metformin therapy, lower age, longer diabetic duration, higher BMI, higher HbA1c, higher blood glucose level were also associated with higher daily insulin dose, and the similar factors were associated with higher insulin doses per weight but different in lower BMI.

In the treatment of type 2 diabetes, β-cell dysfunction and insulin resistance are both associated with higher demand for exogenous insulin [16]. Some factors associated with insulin doses observed in this study were consistent with previous observations, which showed that younger patient’s age, longer diabetic duration, higher HbA1c, higher blood glucose level were associated with higher insulin dose because of poor β-cell function and poor glycemic control [16], [17]. There was a different result in BMI when analyzing associated factors for daily insulin dose and insulin dose per weight, which showed that higher BMI was associated with higher daily insulin dose while lower BMI was associated with higher insulin dose per weight. It could be explained as that patients with higher BMI always have higher body weight, therefore they need more daily insulin dose. But when it turns to insulin dose per weight, patients with lower BMI always have poor β-cell function, therefore, these patients may need more insulin to control glycemic level, but their daily insulin dose might not be high.

According to this study, the mean BD/TDD ratio of Chinese type 2 diabetic patients receiving BBT was 0.23. Of the patients receiving insulin therapy alone or receiving insulin combination with metformin, the mean BD/TDD ratio was 0.22 and 0.24 respectively. The BD/TDD ratio of a large majority (92.45%) of patients was in the range of 0.11–0.40, and the distribution pattern was very similar between patients receiving insulin therapy alone and patients receiving insulin combination with metformin. These results may suggest that these type 2 diabetic patients receiving BBT might remain substantial insulin secretion, therefore, the BD/TDD ratio of these patients was lower than that of type 1 diabetic patients in the Caucasians whose BD/TDD ratio was about 0.50 [18], [19]. This ratio may also be different from that of type 2 diabetic patients in the Europeans and the Americans. In the study that Rosenstock et al reported [6], BD/TDD ratio was about 0.4. In Hollander et al [7] reported study, BD/TDD ratio was about 0.69 in the determir group and 0.65 in the glargine group respectively. In Umpierrez et al [9] reported study, BD/TDD ratio was about 0.53. In Fonceca et al [10] reported study, BD/TDD ratio was about 0.50 in the glargine group and 0.47 in the NPH group respectively. However, in a study reported from Japanese type 2 diabetic patients on intensive insulin therapy [13], the BD/TDD ratio was in accordance with that concluded from our study. In Yokoyama et al [12] reported study, BD/TDD ratio was 0.48. It may suggest that the Asians need less basal insulin for good glucose control.

According to this study, the BD/TDD ratio, was positively associated with age, diabetic duration, BMI, HbA1c, fasting blood glucose level, and using metformin, while negatively associated with postprandial blood glucose level, postprandial C peptide, and CRE level. As we know that, with the increasing of DM duration, the β-cell function decreased, which indicated that patients might need more basal insulin for good glycemic control, therefore, the BD/TDD ratio became higher. Patients included in this study were all type 2 diabetes that characterized as not only insulin secretion deficiency but also insulin resistance. The higher level of BMI indicated the stronger of insulin resistance, and patients with strong insulin resistance may need more basal insulin for good glycemic control. This could also explain why using metformin was associated with higher BD/TDD ratio, for doctors were suggested to use metformin in treating insulin resistance in overweight and obese patients. According to this study, the higher HbA1c or fasting blood glucose level was, the higher BD/TDD ratio was. It is obvious that patients with poor glycemic control may need more basal insulin for good fasting glycemic control, and therefore, BD/TDD ratio was higher. For compared with the younger one, the older patients in this study showed lower BMI and lower HbA1c, therefore, they may need lower BD/TDD ratio for glycemic control. As the C peptide level may reflect the endogenous secretion of β-cell, which could be inhibited by exogenous insulin treatment. So patients receiving more basal insulin dosage might show good glycemic control as lower level of postprandial blood glucose, and might show the inhibited endogenous secretion as lower level of postprandial C peptide. According to this study, the higher the CRE level, the lower the BD/TDD ratio. The average level of CRE was 78.68±40.02 umol/l, which indicated that the renal function of most patients included in this study were normal. However, higher CRE level might influence the insulin metabolism and degradation in patients and therefore might lead to higher risk of hypoglycemia. So for these patients admitted for BBT, doctors prescribed less basal insulin in case of hypoglycemia events. Some of the above factors associated with higher BD/TDD ratio found in this study were consistent with previous observations [16], [17].

The ratios between short acting insulin and intermediate acting protamine of the commercial available premixed preparations are 25∶75, 30∶70 and 50∶50 in current China marketing. However, according to our study which showed that the mean BD/TDD ratio of Chinese type 2 diabetic patients receiving BBT was 0.23, those ratios might not match the real need of insulin therapy in Chinese type 2 diabetic patients. This information might also help clinicians and pharmacologists to choose a better ratio of premixed insulin for individual Chinese patients.

We acknowledge the following limitations in this study. First, this study was a retrospective study, which made it difficult to draw firm conclusions regarding the clinical outcomes, because patients receiving insulin treatment in this study were not originally intended to explore the insulin dose, or the BD/TDD ratio or the associated factors. However, these patients were all admitted patients, and their insulin doses were titrated by experienced doctors according to the insulin treatment protocols. Therefore, results from these admitted patients could help us for better learning insulin regimen in Chinese type 2 diabetic patients. Second, patients of this study were made up of hospitalized patients, so the results could only reflect the factors associated with insulin treatment in admitted patients but not the out-patients.

In summary, from this study, we concluded that average daily insulin dose of Chinese type 2 diabetic patients receiving BBT was 38.22 IU/day (0.58 IU/kg/day). Average basal insulin dose was 8.94 IU/day (0.13 IU/kg/day) and average bolus insulin dose was 29.29 IU/day (0.44 IU/kg/day) and the mean BD/TDD ratio was 0.23.

## References

[1] Nathan DM, Buse JB, Davidson MB, Heine RJ, Holman RR (2006). Management of hyperglycemia in type 2 diabetes: a consensus algorithm for the initiation and adjustment of therapy: a consensus statement from the American Diabetes Association and the European Association for the Study of Diabetes.. Diabetes Care.

[2] Nathan DM, Buse JB, Davidson MB, Heine RJ, Holman RR (2006). Management of hyperglycemia in type 2 diabetes: a consensus algorithm for the initiation and adjustment of therapy: a consensus statement from the American Diabetes Association and the European Association for the Study of Diabetes.. Diabetologia.

[3] Hirsch IB, Bergenstal RM, Parkin CP, Wright E, Buse JB (2005). A real-world approach to insulin therapy in primary care practice.. Clin Diabetes.

[4] Rosenstock J (2004). Basal insulin supplementation in type 2 diabetes: refining the tactics.. Am J Med.

[5] Yki-Jarvinnen H, Dressler A, Ziemen M, HOE 901/300s Study Group (2000). Less nocturnal hypoglycemia and better post-dinner glucose control with bedtime insulin glargine compared with d\bedtime NPH insulin during insulin combination therapy in type 2 diabetes, HOE 901/3002 Study Group.. Diabetes Care.

[6] Rosenstock J, Ahmann AJ, Colon G, Scism-Bacon J, Jiang H (2008). Advancing insulin therapy in type 2 diabetes previously treated with glargine plus oral agents.. Diabetes Care.

[7] Hollander P, Cooper J, Bregnh0j J, Pedersen CB (2008). A 52-Week, Multinational, Open-Label, Parallel-Group, Noninferiority, Treat-to-Target Trial Comparing Insulin Detemir with Insulin Glargine in a Basal-Bolus Regimen with Mealtime Insulin Aspart in Patients with Type 2 Diabetes.. Clinical Therapeutics.

[8] Umpierrez GE, Smiley D, Zisman A, Prieto LM, Palacio A (2007). Randomized Study of Basal-Bolus Insulin Therapy in the Inpatient Management of Patients With Type 2 Diabetes (RABBIT 2 Trial).. Diabetes Care.

[9] Umpierrez GE, Tiffany HT, Smiley D, Temponi A, Umpierrez D (2008). Comparison of Inpatient Insulin Regimens with Detemir plus Aspart versus NPH plus Regular in Medical Patients with Type 2 Diabetes. J Clin Endocrin Metab.. ; 1441.

[10] Fonseca V, Bell DS, Berger S, Thomson S, Mecca TE (2004). A comparison of bedtime insulin glargine with bedtime neutral protamine hagedorn insulin in patients with type 2 diabetes: subgroup analysis of patients taking once-daily insulin in a multicenter, randomized, parallel group study, Am. J. Med. Sci..

[11] Raskin P, Gylvin T, Weng W, Chaykin L (2009). Comparison of insulin detemir and insulin glargine using a basal-bolus regimen in a randomized, controlled clinical study in patients with type 2 diabetes. *Diabetes Metab Res Rev*..

[12] Yokoyama H, Tada J, Kamikawa F, Kanno S, Yokota Y (2006). Efficacy of conversion from bedtime NPH insulin to morning insulin glargine in type 2 diabetic patients on basal-prandial insulin therapy, Diabetes Res. Clin. Pract.. 73: () 35–40.

[13] Tamaki M, Shimizu T, Kanazawa A, Fujitani Y, Watada H (2008). Effects of changes in basal/total daily insulin ratio in type 2 diabetes patients on intensive insulin therapy including insulin glargine (JUN-LAN Study 6). Diabetes Research and Clinical Practice..

[14] Masuda H, Sakamoto M, Irie J, Kitaoka A, Shiono K (2008). Comparison of twice-daily injections of biphasic insulin lispro and basal-bolus therapy: glycaemic control and quality-of-life of insulin-naïve type 2 diabetic patients. Diabetes, Obesity and Metabolism..

[15] Miyashita Y, Nishimura R, Nemoto M, Matsudaira T, Kurata H (2008). Prospective randomized study for optimal insulin therapy in type 2 diabetic patients with secondary failure. Cardiovascular Diabetology..

[16] UK Prospective Diabetes Study (UKPDS) Group (1998). Intensive blood-glucose control with sulphonylureas or insulin compared with conventional treatment and risk of complications in patients with type 2 diabetes (UKPDS 33) [published correction appears in Lancet. 1999;354:602]. Lancet..

[17] Goudswaard AN, Furlong NJ, Valk GD, Stolk RP, Rutten GE (2004). Insulin monotherapy versus combinations of insulin with oral hypoglycaemic agents in patients with type 2 diabetes mellitus. Cochrane Database Syst Rev.. Oct 18.

[18] The Diabetes Control and Complications Trial Research Group (1993). The effect of intensive treatment of diabetes on the development and progression of long-term complications in insulin-dependent diabetes mellitus. N Engl Med J..

[19] Vague P, Selam JL, Skeie S, Leeuw ID, Elte JWF (2003). InsulinDetemir Is Associated With More Predictable Glycemic Control and Reduced Risk of Hypoglycemia Than NPH Insulin in Patients With Type 1 Diabetes on a Basal-Bolus Regimen With Premeal Insulin Aspart. Diabetes Care..

